# Social Activities in Community Settings: Impact of COVID-19 and Technology Solutions

**DOI:** 10.1093/geroni/igaa057.3499

**Published:** 2020-12-16

**Authors:** Anne Adams, Jenay Beer, Xian Wu, Jane Komsky, Jason Zamer

**Affiliations:** 1 SimpleC, LLC, Atlanta, Georgia, United States; 2 Institute of Gerontology, Athens, Georgia, United States; 3 University of Georgia, Athen, Georgia, United States

## Abstract

COVID-19 has created challenges for staff in promoting resident activity. To better understand the pandemic-related challenges that Activity Professionals are facing, we asked COVID-19 specific questions as part of a larger survey. The overall survey focused on identifying challenges and potential technology solutions (e.g., socially assistive robots) to assist Activity Professionals in their job duties. Activity Professionals (N=19) completed the online questionnaire. Respondents (aged M=48.00, SD=12.87; 95% female, 100% native English speakers, 68% White/Caucasian, 21% Black/African American) were highly educated/experienced: 68% had a Bachelor’s degree or above, and 53% had 10-35 years of experience. Respondents worked in Independent Living (68%), Assisted Living (37%), Memory Care (26%), Skilled Nursing (21%), or Personal Care (11%). All Activity Professionals reported impact by COVID-19, as follows: 1) Cancelled activities: Group activities/gatherings; hosting outside entertainment; fewer volunteers, vendors, paid sources. 2) New restrictions: Number of people in elevators, rooms; no contact with residents. 3) Unexpected new tasks: More 1:1 meetings; video conferencing; additional phone calls with residents, staff, and families; ordering groceries online. Daily duties changed significantly with less help and limited availability of technology. 4) Effect: Fewer activities and new delivery models (online, TV). Concerns about potential negative effects on residents while trying to meet the creativity challenge: “This has caused lots of “out of the box” thinking for ways to engage residents and keep things upbeat despite the challenges.” Results illustrate the breadth of challenges that staff are facing, some of which can be addressed by technology.

